# Quantification of mid and late evoked sinks in laminar current source density profiles of columns in the primary auditory cortex

**DOI:** 10.3389/fncir.2015.00052

**Published:** 2015-10-02

**Authors:** Markus K. Schaefer, Julio C. Hechavarría, Manfred Kössl

**Affiliations:** Institute for Cell Biology and Neuroscience, AK Neurobiology and Biosensors, Goethe UniversityFrankfurt/Main, Germany

**Keywords:** local field potentials, current source density, late evoked potentials, intracortical network processing, spectral integration, horizontal projections, cortical layers, primary auditory cortex

## Abstract

Current source density (CSD) analysis assesses spatiotemporal synaptic activations at somatic and/or dendritic levels in the form of depolarizing current sinks. Whereas many studies have focused on the short (<50 ms) latency sinks, associated with thalamocortical projections, sinks with longer latencies have received less attention. Here, we analyzed laminar CSD patterns for the first 600 ms after stimulus onset in the primary auditory cortex of Mongolian gerbils. By applying an algorithm for contour calculation, three distinct mid and four late evoked sinks were identified in layers I, III, Va, VIa, and VIb. Our results further showed that the patterns of intracortical information-flow remained qualitatively similar for low and for high sound pressure level stimuli at the characteristic frequency (CF) as well as for stimuli ± 1 octave from CF. There were, however, differences associated with the strength, vertical extent, onset latency, and duration of the sinks for the four stimulation paradigms used. Stimuli one octave above the most sensitive frequency evoked a new, and quite reliable, sink in layer Va whereas low level stimulation led to the disappearance of the layer VIb sink. These data indicate the presence of input sources specifically activated in response to level and/or frequency parameters. Furthermore, spectral integration above vs. below the CF of neurons is asymmetric as illustrated by CSD profiles. These results are important because synaptic feedback associated with mid and late sinks—beginning at 50 ms post stimulus latency—is likely crucial for response modulation resulting from higher order processes like memory, learning or cognitive control.

## Introduction

Population activity of neurons in the mammalian cortex and their interaction with subcortical as well as with other cortical regions can be evaluated using local field potentials (LFP). These summed potentials are thought to reflect synaptic activity and the sequence of characteristic LFP waves is considered to indicate distinct stages in the cortical processing of information (Barth and Di, 1991; Medvedev and Kanwal, 2004). Studies on the differential effects of anesthesia (Barth and Di, 1990), sleep (Knight et al., 1985), and alertness (Satya-Murti et al., 1983; Knight et al., 1985) on the amplitudes and spatiotemporal distribution of LFP peaks, gave evidence that these potentials may be generated by multiple brain regions. In the auditory cortex, the early positive/negative biphasic wave (P1/N1), which is commonly found in rats (Barth and Di, 1990; Simpson and Knight, 1993; Takahashi et al., 2007), guinea pigs (Kraus et al., 1985), gerbils (Ohl et al., 2000), and cats (Kaga et al., 1980), most likely reflects depolarization triggered by thalamic or intracortical inputs (Kaga et al., 1980; Barth and Di, 1990; Di and Barth, 1992; Thomas et al., 1993; Ohl et al., 2000; Kaur et al., 2004).

Successive waves are proposed to be generated by different areas of the primary and secondary cortical fields, the frontal cortex (FC), the centro-parietal cortex, association cortex or hippocampus (Picton et al., 1974; Skrebitsky and Sharonova, 1976; Kraus et al., 1985; Näätänen and Picton, 1987; Barth and Di, 1990; Santos Filha and Matas, 2010).

However, single LFP recordings representing the sum of inhibitory and excitatory synaptic activity cannot provide precise spatial information about a layer- or depth-specific location of various activation components, although they have been reported to induce lower thresholds for responses to tone bursts than for simultaneously recorded spiking activity (Galvan et al., 2002). The current source density (CSD) analysis, which is based on the second spatial derivative of the field potentials along the radial depth, localizes synaptic inputs. This method provides precise spatial and temporal information about the functional weights of synaptic activity (sinks) and therefore points to the mechanism of their generation. It thus allows to trace the neuronal information flow (Nicholson and Freeman, 1975; Mitzdorf, 1985).

Studies using CSD analysis in combination with cortical silencing showed that initial sinks originate from thalamic or intracortical regions (Kaur et al., 2005; Happel et al., 2010). The subsequent sinks were thus hypothesized to be evoked through transynaptic intracortical processing. Repetitive after-discharges originating from the thalamus or inputs from outside of the AC such as the FC, the contralateral hemisphere or the hippocampus are also possible generators (Rappelsberger et al., 1981; Mitzdorf, 1985). Mid and late sinks could be crucial for understanding higher order processes like memory, learning or cognitive control and provide the basis for modeling precise neuronal circuits.

Several studies on early and mid sinks proposed that intracortical connections are responsible for spectrally distant inputs (Kaur et al., 2004, 2005; Tomioka et al., 2005; Kurt et al., 2008; Happel et al., 2010; Moeller et al., 2010). In contrast, low level sounds are believed being temporally integrated by the pressure envelope of the sound (Heil and Neubauer, 2001, 2003; Heil et al., 2008). It would be interesting to compare both integration mechanisms at the cortical level. To our knowledge, studies on level and spectral integration of late sinks have not yet been done. Differences in these sinks evoked by stimuli at the borders of receptive fields could be a result of stimulus-specific proportioned spatial and temporal interactions and convergences and thus help to complete the understanding of these integration mechanisms.

To study the differences, origins, laminar locations and temporal characteristics of sinks, we qualitatively and quantitatively characterized the mid and late evoked current sinks (50–575 ms post stimulus) evoked by stimuli at the borders of receptive fields in the primary auditory cortex of Mongolian gerbils. We could identify three mid and four late evoked sinks, which were less reliably evoked compared to the initial two sinks in layers III/IV and V/VI. The high resolution of laminar profiles allowed us to propose a hypothetical scheme of the specific sink generators based on results of previous studies and on the cortical architecture. Although the intracortical information-flow patterns remained qualitatively similar for the stimuli, we could find differences in the strength, vertical extent, onset latency, and duration of the sinks, which could be caused by either temporal or spectral integration. The appearance of a new sink emerged in layer Va when a stimulus one octave above the characteristic frequency (CF) was tested and the disappearance of the layer VIb sink at low level stimulation indicated the presence of input sources specifically activated in dependence of level and/or frequency. Our results further indicated an asymmetry regarding synaptic inputs associated with low vs. high frequency processing in the cortex.

## Materials and Methods

The study was carried out in 8 adult Mongolian gerbils (*Meriones unguiculatus*; age: 6–10 month; body weight: 59–79 g) of both sexes (five females and three males). Animals were taken from a breeding colony in the Institute for Cell Biology and Neuroscience, Goethe University, Frankfurt am Main, Germany. All experiments were conducted in accordance with the regulations by the International National Institutes of Health Guidelines for Animals in Research and with ethical standards for the care and use of animals in research defined by German Law for the protection of experimental animals (Experimental permit # F104/60).

### Surgical Procedures

All animals were initially anesthetized with a mixture of ketamine (100 mg/kg; Ketavet, Pfizer, New York, USA), xylazine (20 mg/kg; Rompun, 2%; BayerVital, Berlin, Germany) and isotonic sodium chloride solution (100 mg/kg; 0.9%; B. Braun, Melsungen, Germany). Anesthesia was maintained with the same mixture throughout the whole experiment with an injection pump (flow-rate: 75 mg/kg/h, Genie, Kent Scientific Corporation, Torrington, CT, USA) while regularly monitoring the hindlimb withdrawal reflex and whisker activity. Body temperature was kept at 37°C using a thermostatic heating blanket. After removing the *musculus temporalis*, the skull was cleaned and a custom-made metal rod (1 cm length, 0.3 cm diameter) was glued onto it using dental cement (Paladur; Heraeus Kulzer, Hanau, Germany). A craniotomy (~3 × 3 mm) was made using a drilling device to expose the left auditory cortex. The opening was cleaned from bone splints and the *dura mater* was completely removed with an injection needle.

### Electrophysiological Recordings

Recordings were performed in a custom-built sound-proof and electrically-shielded chamber. The neuronal activity was recorded using commercially available linear probes (Model: A1 × 16-3 mm-100-177-A16, NeuroNexus, Ann Arbor, MI, USA) with 16 contacts (impedance: 0.5–3 MΩ; spacing: 100 μm) spanning 1500 μm. Such electrode design is well suited for obtaining recordings from entire cortical columns, which span ~1300 μm in the Mongolian gerbil (Sugimoto et al., 1997). Using a micro-manipulator system (PM 10/1, Science Products GmbH, Hofheim, Germany), electrodes were inserted slowly into the brain (20 μm/s) and placed perpendicular to the pial surface at a depth of ~1500 μm, so that the top channel was located above the cortical surface. Orthogonality between electrode and surface was controlled by adjusting the animal’s head several times until the altitude differences between the four corners of the temporal hole were <70 μm utilizing the electrode as an altimeter. Using this technique once for each animal, the characteristic frequencies of neurons recorded at 400–1000 μm (layers IV–VIa) of each track (6.4 ± 3.9 tracks per animal) were constant within a range of ±0.34 octaves.

Layer depth localization was adopted from a previous study by Sugimoto et al. (1997): layer I was located at 0–120 μm, layer II at 120–210 μm, layer III at 210–410 μm, layer IV at 410–560 μm, layer V at 560–850 μm, and layer VI at 850–1300 μm depth from the cortical surface. Neuronal activity was preamplified (10×, μPA16, Multichannel Systems, Reutlingen, Germany) and recorded using a multichannel recording system (amplification: 1000×, ME32, Multichannel Systems, Reutlingen, Germany). To obtain LFP, recorded signals were digitally bandpass-filtered offline between 0.2 and 300 Hz (butterworth, 2nd order) and notch-filtered at 50 Hz to remove humming noise, downsampled from 50 to 20 kHz and stored in a computer for further analysis.

### Acoustic Stimulation

Pure tones were digitally synthesized and controlled using a custom-written program in Matlab (R2007b, MathWorks, Natick, MA, USA). Stimuli were generated by an external soundcard (e18 dac, exaSound, Toronto, Canada, sampling rate: 192 kHz), amplified (RB-1050, Rotel Electronics, Tokyo, Japan) and delivered from a calibrated speaker (SS-MS835, Sony, Tokyo, Japan). The calibration curve was obtained with a Brüel and Kjaer sound recording system (¼-inch Microphone 4135, Microphone Preamplifier 2670, Brüel and Kjaer, Naerum, Denmark) connected to a conditioning microphone amplifier (Nexus 2690, Brüel and Kjaer, Naerum, Denmark). During the experiment, the speaker was placed in front of the animal’s right ear at a distance of 20 cm. We presented the animal with a pseudorandomized series of pure tones at different intensities (step size: 10 dB, range: 0–80 dB SPL) with either a logarithmic or linear frequency spacing (step size: 0.5 oct/3.5 kHz, range: 0.25–64 kHz/0.5–56.5 kHz), depending on the respective neuronal receptive field (borderline at 11 kHz). We calculated neuronal tuning curves for a threshold value of 30% of the maximum spiking rate. The CF was defined as the stimulus frequency that elicited a response at the minimum threshold (MT) of the tuning curve. The CF was calculated from the neuronal response obtained in layer V/VI, and for the present data set only penetrations that yielded sensitive (MT <50 dB SPL) and V-shaped tuning curves in accordance with the tonotopic map of gerbils (Thomas et al., 1993) were considered. The response to four stimuli combinations was used for analyzing columnar CSD patterns: (1) pure tone at CF at 80 dB SPL (from now on CF80); (2) pure tone one octave below the CF at 80 dB SPL (from now on −1oct); (3) pure tone one octave above the CF at 80 dB SPL (from now on +1oct); and (4) pure tone at the CF ≤24 dB above the individual MT (from now on CF24+). The level of the latter was conditioned by the measuring level step size of 24 dB. All stimuli were presented in pseudorandomized order and sampled at 192 kHz. The duration was 30 ms with a 5 ms rise-fall. The interstimulus interval was set at 0.6 s.

### Current Source Density Analysis

**Figure 1:**
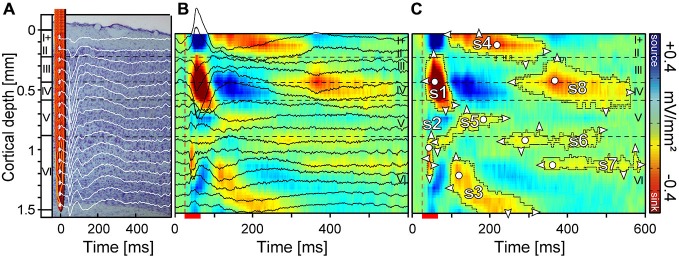
**Laminar local field potentials (LFP) and current source density (CSD) profile of a primary auditory cortex site tuned to 5.7 kHz. (A)** Field potentials were recorded with a linear-array multicontact electrode covering all six cortical layers. Recordings were obtained simultaneously at each depth at an interchannel distance of 100 μm. **(B)** CSD profiles were calculated from field potentials with color indicating current strength and direction. Current sinks (red) are classically interpreted as net inward transmembrane currents and current sources (blue) are net outward currents. **(C)** For the quantification of sinks, several contour lines were plotted using a criterion of 8% of the maximum sink strength. Eight sinks (s1–s8) could be defined within a time window of 600 ms. White circles represent the location of the maximum strength and white triangles the horizontal and vertical dimensions. Horizontal dashed lines indicate layer borders while the vertical dashed line marks the beginning of pure tone stimulation. Red scale bar represents pure tone stimulus duration of 30 ms.

Neuronal activity was recorded simultaneously from all cortical layers of 51 penetration sites in the left primary auditory cortex. Bandpass-filtered LFPs were averaged over 25 stimulus repetitions (Figure 1A). The standard CSD method assumes a homogeneous activity along the horizontal direction and uses a discretized version of the Poisson’s equation. It further assumes that the extracellular medium acts as a volume conductor that is ohmic at the relevant frequency range (Nicholson and Freeman, 1975; Mitzdorf, 1985; Pettersen et al., 2006; Szymanski et al., 2009). We calculated one-dimensional CSD profiles from the second spatial derivative of the LFP, which can be approximated using the following formula:

$$\frac{\delta^{\text{2}}\phi }{\delta {\text{z}}^{\text{2}}}\approx \frac{\phi \left({\text{z}}_{\text{0}}\;\text{+}\;\text{n}\Delta \text{z}\right)\;\text{+}\;\phi \left({\text{z}}_{\text{0}}\;\text{-}\;\text{n}\Delta \text{z}\right)\text{-}\;\text{2}\phi \left({\text{z}}_{\text{0}}\right)}{{\left(\text{n}\Delta \text{z}\right)}^{\text{2}}}$$

The double of the field potential (φ) at the cortical depth z_0_ is subtracted from the summated adjacent field potentials above (z_0_ + nΔz) and below (z_0_ − nΔz) the field potentials at depth z_0_ (interchannel distance Δz = 100 μm) and divided by the differentiation grid (nΔz; *n* = 1). For the CSD calculation, a modified version of the iCSDplotter toolbox was used (Pettersen et al., 2006). Estimates for the CSD at top and bottom electrode channels were provided by the method of Vaknin et al. (1988). To reduce spatial noise, a three-point Hamming filter was applied (Rappelsberger et al., 1981):

$$\phi_{\text{filt}}\left(\text{z}\right)\text{=0}.\text{23}\phi \left({\text{z}}_{\text{0}}\;\text{+}\;\text{n}\Delta \text{z}\right)\;\text{+}\;\text{0}.\text{23}\phi \left({\text{z}}_{\text{0}}\;\text{-}\;\text{n}\Delta \text{z}\right)\;\text{+}\;\text{0}.\text{54}\phi \left({\text{z}}_{\text{0}}\right)$$

In the resulting laminar CSD profiles (Figure 1B), current sinks are classically interpreted to indicate excitatory events e.g., axonal depolarizations and excitatory or inhibitory synaptic activations and current sources are in most cases the passive return currents (Mitzdorf, 1985). Visualization of laminar profiles was improved by linear channel interpolation.

### Contour Calculation and Parameter Quantification of Sinks

Accurately determining the onset and offset latencies of sinks has been described as difficult in previous studies (Kaur et al., 2004; Happel et al., 2010). Here, to obtain global criteria for separating individual sinks, we used a two step calculation method. In the first step, contours were plotted around all sinks of a laminar profile, which surpassed 8% of the maximum sink amplitude of the CSD profile (Figure 1C) elicited with CF80 stimulation of the respective column. Contours were calculated using Matlab’s contour function. In the second step, contour depending parameters of sinks such as area size, onset latency, duration, vertical extent, maximum strength and its depth were automatically calculated with custom-written programs using Matlab. Resulting sink patches were merged together if the temporal distance accounted for less than 25 ms. Fusions between sinks were manually separated at their narrowest point in accordance with the sink structure of the averaged CSD patterns. In the result section we focus on s1, s3, s5, and s8 as we assumed that analyzing those sinks could be sufficient to quantitatively access the stimulus-specific characteristics of sinks and layer dependent differences. In our analysis, s3 was of special interest, as its activation spanned over a large area within layer VI likely involving different types of pyramidal cells projecting to thalamic nuclei. Sink s5 was chosen, despite of its small area of activation, as it was reliably evoked and it is the only sink in layer V which is known to receive input from subcortical (medial division of the geniculate body) and contralateral areas such as the frontal, entorhinal and auditory cortex (Linden and Schreiner, 2003). Sink s8 had the largest area compared to all remaining sinks and its location in the main thalamic input layers III/IV suggests an important role for the integration of ispilateral and contralateral inputs (Linden and Schreiner, 2003).

### Statistical Analysis

Data of sink parameters were tested for normal distribution with a Kolmogorov–Smirnov test. A non-parametric one–way analysis of variance (Kruskal-Wallis) was then applied in combination with a multiple comparison *post hoc* test in Matlab (multcompare function). All statistical analysis was performed with custom-written programs using Matlab. Tests that rendered *p* values <0.05 were considered as significant.

### Recording Site and Map Construction

**Figure 2:**
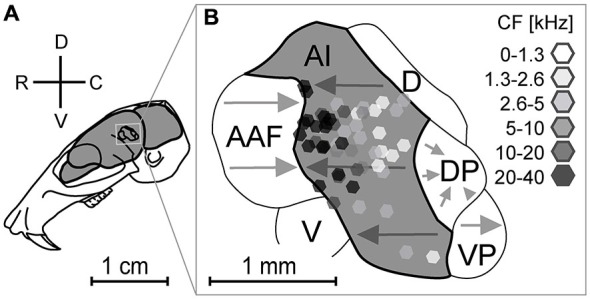
**Location of recording sites in relation to the functional organization of the auditory cortex.** Location **(A)** and schematic representation of the parcellation of the auditory cortex **(B)**. Four subdivisions, AI (primary auditory cortex), AAF (anterior auditory field), VP (ventroposterior field), and DP (dorsoposterior field) show tonotopically organized neurons (gray arrows). The dorsal field (D) and ventral field (V) are not tonotopically organized. All laminar tracks (*n* = 51, octagons) were recorded within the AI. Grayscale colors of octagons give the characteristic frequencies. Arrowheads point towards higher frequencies. Adapted from Thomas et al. (1993) and Budinger et al. (2000).

The location and size of the gerbil’s AC is schematically represented in Figure 2A. Several functional areas (Figure 2B) were identified in previous studies (Thomas et al., 1993; Budinger et al., 2000, 2008). All recording locations (Figure 2B) were verified by using the stereotaxic coordinates of penetration points and the suture intersection of parietal, sphenoidal and temporal bone as reference. The course of blood vessels was variable and therefore it could not be used for localization of penetrations. We chose the recording sites to obtain an evenly distributed representation of neurons in the AI at the planar and laminar level. CFs of the studied recording points ranged between 0.5 and 28 kHz, with 24 penetrations above 10 kHz and 27 below this value.

## Results

LFPs were measured along 51 penetrations in the left primary auditory cortex (AI) of eight adult Mongolian gerbils, while presenting a set of four stimuli differing in frequency and level. The neuronal signal was recorded in parallel from 16 equally spaced (100 μm) channels from the *pia mater* to the white matter. Using the LFPs, one dimensional CSD patterns were calculated. The CSDs are essentially an ensemble activity mainly caused by excitatory synapses. The vertical extent in depth of each sink and its corresponding source (or sources) indicates the region over which the activated cells extend their dendrites (Mitzdorf, 1985). In the following, we characterize the current sinks with focus on the mid and late evoked components and quantify the changes in the CSD patterns to spectrally distant and suprathreshold stimuli, to assess their relative contributions to the responses’ temporal structure.

### Frequency Representation of Neurons in the AI

**Figure 3:**
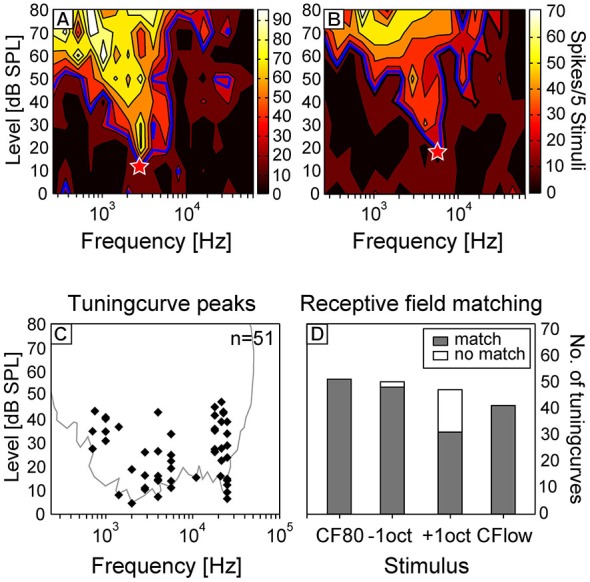
**Analysis of receptive fields of cortical neurons.** Representative tuning curves of two neurons of different animals are displayed in the upper row [characteristic frequency (CF; red asterisks) = 2.8 kHz **(A)**; 5.7 kHz **(B)**]. Tuning curves were interpolated at 30% of the maximum spike rate. **(C)** CF displayed against minimum threshold (MT) of all tuning curves measured at layer V/VI of each cortical site. In the background the outline of all shapes of respective receptive field is plotted. Only neurons with sensitive receptive fields (MT below 50 dB SPL) were selected for this study. **(D)** The percentage of receptive fields matching with the respective frequency content of the four stimuli.

To investigate the current sinks that appeared in response to different stimuli, we first characterized the frequency tuning of each recording site by presenting pure tones of varying frequency and intensity. The neurons of the tonotopically organized core area AI are known to respond to narrow frequency ranges with short latencies of ~10–25 ms (Thomas et al., 1993) and receive cochleotopic input directly from the ventral and medial division of the medial geniculate body (Wessinger et al., 2001; Linden and Schreiner, 2003). Due to a columnar cortex organization, the neuronal CF remains relatively similar in each layer (Wallace and Palmer, 2008) and therefore we considered it sufficient for the characterization of the entire penetration site to analyze the tuning curve shape and CF at one depth. Two representative tuning curves of different animals are displayed in Figures 3A,B. Tuning curves showed characteristic V-shapes with higher spiking activity at increasing sound pressure levels. The CF (red asterisks) at the peak of the tuning curves, which were interpolated at 30% of the maximum spiking rate, were calculated for each electrode penetration at layer V/VI. In this layer stimuli elicited the shortest onset latencies for sink s2 (Figure 1C). The frequency of the CF80, the 80 dB SPL pure tone at which the CSD patterns were analyzed, was adjusted in a way that it matched the CF calculated from the tuning curves calculated for layer V/VI.

In Figure 3C the CF is displayed against the MT of all tuning curves measured in layer V/VI. In gray, the global outline of all shapes of respective receptive fields is plotted. The shape of this threshold curve corresponds to the behavioral hearing threshold curve of Ryan (1976), but thresholds were about 5–15 dB less sensitive than in the behavioral data. The hearing-sensitivity-decrease between 5 and 15 kHz was more pronounced in the present study and was probably due to the sparse distribution of neurons having their CF at this frequency range. The matching of the frequency of the four stimuli with the respective receptive fields is displayed in Figure 3D. As one would expect, in 100% of the cases, the receptive fields of the studied neurons overlapped with the frequencies of CF80 and CF24+ stimuli. The frequency of −1oct and +1oct matched in 96 and 66% with the respective receptive field. In most cases (81%), in which the frequency content of +1oct appeared outside of the receptive fields, the CF was found to be above 20 kHz which drove the frequency of +1oct to the upper end of the animals hearing range.

### Changes of CSD Patterns to Different Stimuli

In our study, we could identify two initial (s1 and s2), three mid (s3, s4, and s5), three late evoked sinks (s6, s7, and s8, see Figure 1C; colored in red), and one late sink (s9) appearing in a few cases within a 600 ms recording window. The initial sinks s1 and s2 characterized by the shortest latencies were located in the thalamic input layers III/IV and V/VI and from now on they will be referred to as primary sinks. Primary sinks appeared to be the most prominent and most reliably evoked components in the CSD patterns observed across the analyzed stimuli. The secondary sinks (s3–s8) were more variable and less reliably evoked across laminar profiles.

**Figure 4:**
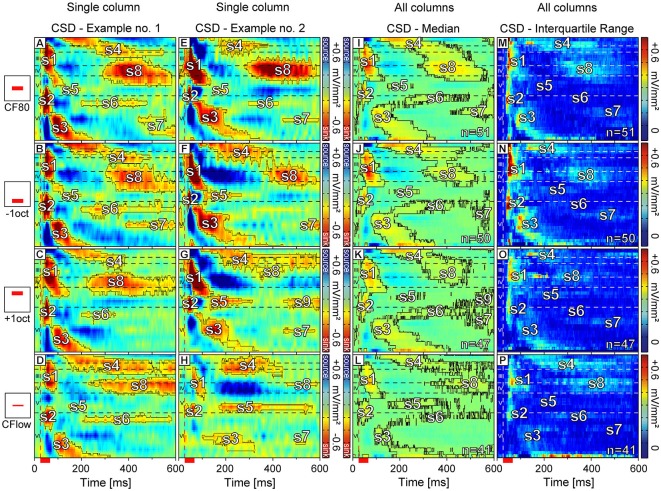
**Color maps of representative and averaged laminar CSD profiles.** In the first and second column **(A–H)** representative CSD profiles of the same cortical column are shown. Neurons were stimulated with four different stimuli: sinus at CF at 80 dB SPL **(A,E)**, sinus at CF one octave below **(B,F)**, sinus at CF one octave above **(C,G)**, and sinus at CF with a level set ≤24 dB above the individual MT **(D,H)**. Sink contours were calculated at 8% of the maximum sink strength of the respective CSD pattern elicited by CF80 stimulation. **(I–L)** The median of laminar profiles averaged over all recording sites for each stimulus respectively. Sink contours were calculated at 4% of the maximum sink strength of the respective CSD pattern elicited by CF80 stimulation. **(M–P)** The interquartile range of sinks for each stimulus group specifically. Horizontal dashed lines indicate layer borders while the vertical dashed line marks the beginning of pure tone stimulation. Red scale bar represents stimulus duration of 30 ms.

Two examples of CSD patterns of different animals obtained with different stimuli at two cortical sites are displayed in Figures 4A–H. The corresponding tuning curves interpolated from spike data at 950 μm depth (layer VI; CF = 2.8 kHz; 5.7 kHz) are displayed in Figures 3A,B. Sink contours were calculated at 8% of the maximum sink strength of the respective CSD pattern elicited by CF80 stimulation. The examples in Figures 4A–H illustrate the variability observed in the CSD patterns. Although there were differences between the two sets of CSD patterns, they were qualitatively very similar and could be interpreted as reflecting the same basic pattern of excitatory synaptic activations. Over all recording sites, the earliest sink of CF80 stimulation (the one with the shortest latency) was s2 [11.7 ± 2.7 ms (mean ± SD)] which appeared at a depth of 867 ± 254 μm (mean ± SD) followed by sink s1 [15.5 ± 4.6 ms (mean ± SD)] with a small delay [4 ± 3.9 ms (mean ± SD)] at a depth of 320 ± 119 μm in layer IV. Secondary sinks emerged in layer V (s5), layer VI (s3), and layer I (s4). The sink s6 followed with a short delay in layer VIa. Latest sinks arose in thalamic input layers III and IV (s8) and layer VI (s7). Some primary sinks of cortical sites [s1 (23%); s2 (47%)] showed pre-discharges which were artifacts produced by the digital notch-filter and were thus excluded.

CSD patterns emerging from +1oct (Figures 4C,G) and CF24+ (Figures 4D,H) had in comparison to patterns emerging from CF80 (Figures 4A,E) and −1oct (Figures 4B,F) lower strength for s1–s4 and s8. The strength of s5–s7 remained relatively low independent of the applied stimulus. The patterns evoked by CF80 and −1oct shared similarities concerning the sink shape and duration for s1–s6. Patterns elicited by CF24+ stimulation showed a rather different structure, when compared to all other stimuli (Figures 4D,H). Primary sinks had longer onset latencies [s1 delay: 24.29 ± 11.3 ms; s2 delay: 19.3 ± 11.6 ms (mean ± SD)] and secondary sinks s4, s5, and s6 –if present– were prolonged in duration in comparison to responses to CF80 [s1 delay: 15.5 ± 4.6 ms; s2 delay: 11.7 ± 2.7 ms (mean ± SD)]. In the CSD profile arising from +1oct stimulation (Figure 4G) a new sink s9 appeared that was located in layer V. This sink was spatially separated from s5 and there was no clear trend that s9 could be associated with specific CFs or MTs. In some profiles (see Figures 4G,H) +1oct and CF24+ stimulation evoked s8 sinks (defined as s8 only if clearly separated from s4) with their point of maximum activity shifted towards layer III. Remarkably, s7 was often missing or the sink strength did not surpass the applied criterion (Figures 4C,D). The overall variability regarding the presence and the area size was increased especially for the secondary sinks.

The exemplary CSD patterns shown in Figures 4A–H reflect the data at the population level (Figures 4I–L). All recordings were aligned in depth and merged together for each stimulus respectively. Each point in the pattern represents the median value. In contrast to the individual examples of CSD patterns (Figures 4A–H) the sink contours were calculated at 4% of the maximum sink strength of the median CSD pattern evoked by CF80 stimulation (Figure 4I). This criterion had to be applied as the median calculation lead to a reduced sink and source strength due to the relatively high variance of CSD patterns. Sink s7 was faintly present (Figures 4J–L), likely due to its unreliable nature in individual laminar profiles. For +1oct stimuli (Figure 4K), sink s9 appeared also at the population level with a relatively strong maximum strength in layer V.

As mentioned before, CSD patterns even within the same stimulus group were relatively variable. To account for these variances and to quantify the extent and location of the different sinks, we calculated the interquartile range exclusively for negative values (sinks) of each stimulus group specifically (Figures 4M–P). The highest variances could be observed for sinks s1, s2, and s3 in the −1oct patterns. These are also known to be the most prominent sinks regarding their strength and also showed relatively high variances for the other stimuli. While the s5 variances evoked by CF80 remained low, they were more pronounced for −1oct and +1oct. Sink s6 showed the highest variances during CF24+ stimulation. The variances for s4, s7, and s8 were relatively small for all stimulus groups.

### Reliability of Evoked Sinks

**Figure 5:**
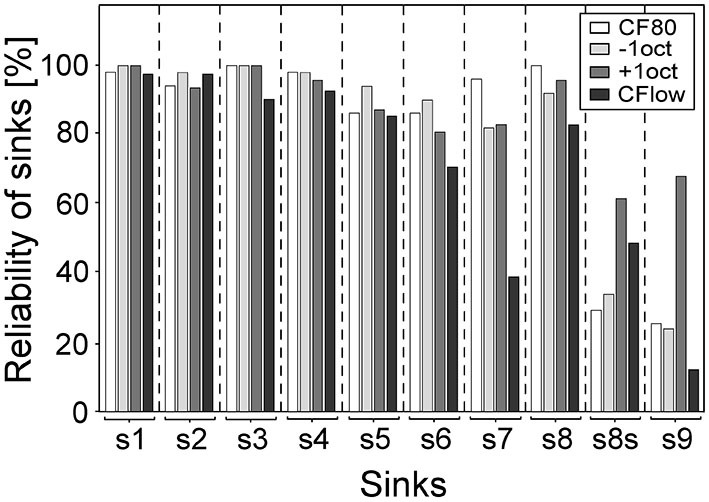
**Reliability of sinks.** Bars give the percentage of presence of sinks for the different stimuli. Sinks s1–s4 and s8 were present in most of the laminar profiles (mean = 96%). Sink s7 of CF24+ was absent in 61% of profiles. The maximum activity of s8 (s8s) elicited by +1oct and CF24+ was located in 62 and 49% of laminar profiles above the layer III/IV border. The sink s9 of +1oct was present in 68% of the laminar profiles.

As the variances between laminar profiles are dependent on the occurrence of sinks, the percentage of sinks for the different stimulations is displayed in Figure 5. The most reliably evoked sinks were primary sinks s1 and s2 and secondary sinks s3, s4, and s8 (mean = 96%). Sinks s5 and s6 were less reliably evoked and appeared in 86% of profiles. Sink s7 at CF80, −1oct and +oct was present at the same level as s5 and s6 but only present in 39% for CF24+ stimuli. The laminar depth of the maximum activity of s8 (s8s) elicited by +1oct and CF24+ was located in 62 and 49% of laminar profiles in layer III, respectively. This shift was present in 29% in CF80 and in 34% in −1oct. The sink s9, which was rarely present for −1oct, CF80, and CF24+ (mean = 21%), was strongly present in 68% of profiles for +1oct stimuli. Regarding s1–s8, CF80 stimulation led to the most stable evoked sinks with an average occurrence-rate of 95%, followed by −1oct (94%), +1oct (92%) and CF24+ (82%).

### Quantitative Analysis of Sink Parameters

To quantify the differences of the stimulus-specific CSD profiles we plotted contours around the sinks of laminar profiles, which surpassed 8% of the maximum sink amplitude elicited by CF80 and calculated the maximum strength, onset latency, duration and vertical extent of each sink. In the following, we focus on s1, s3, s5, and s8 and present the changes between CF80, −1oct, +1oct, and CF24+ stimulation. We chose the aforementioned sinks because they represent the primary thalamocortical input (sink s1), and the strongest secondary sinks of granular layer IV (s8), infragranular layer V (s5) and VI (s3). We assumed that analyzing those sinks could be enough to quantitatively access the stimulus-specific characteristics of sinks and layer dependent differences. A more detailed analysis of the differences between all sinks can be found in the supplementary information (Supplementary Figure 1).

**Figure 6:**
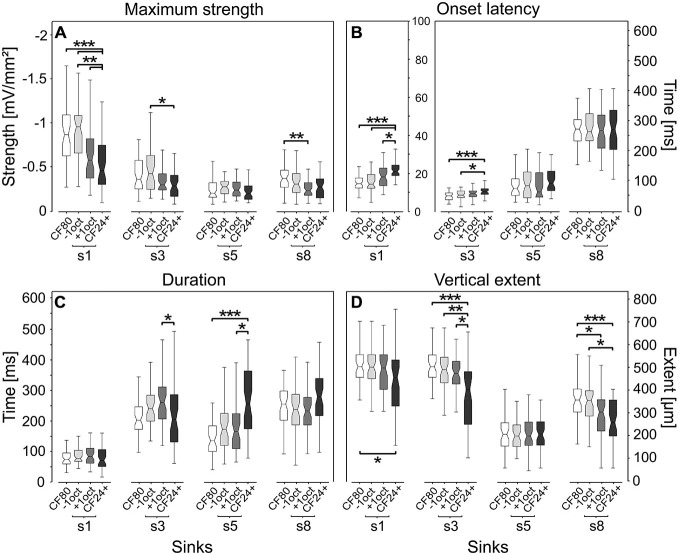
**Quantitative comparison of sink parameters.** Parameters were calculated at 8% of the maximum sink strength of the respective CSD pattern elicited by CF80 stimulation. Low level pure tones (CF24+) and pure tone frequencies of one octave distant from CF (−1oct and +1oct) evoke significantly different sinks. Primary sink s1 (see sinks schematic above) and the most strongly elicited secondary sinks from infragranular layer VI (s3) and V (s5) and granular layer IV (s8) are compared. Four parameters were quantitatively assessed: **(A)** maximum sink strength, related to the strength of neuronal activity. **(B)** Sink onset latency, note the different y-axes for s1 vs. other sinks. **(C)** Sink duration. **(D)** Sink vertical extent. Boxplot whiskers represent data range, outer edge of box represents second and fourth quartiles of data, and midline represents median of data. Significance was determined using Kruskal-Wallis one-way ANOVA in combination with a multiple comparison *post hoc* test: *p < 0.05, **p < 0.01, ***p < 0.001.

We found low level pure tones (CF24+) and pure tone frequencies of one octave distant from CF (−1oct and +1oct) to evoke significantly different secondary sinks compared to CF80, indicating that different stimuli activate different cortical circuits. The maximum sink strength (Figure 6A) showed a relatively comparable pattern for s1, s3 and s8, in which CF80 and −1oct mostly elicited significantly higher maximum sink strengths when compared to +1oct and CF24+. Similar maximum sink strengths for s1 (–1.11 mV/mm^2^) could be evoked with best frequency stimuli by Happel et al. (2010). Sink s5 showed a completely different behavior with non-significant differences and relatively comparable values across analyzed stimuli. This is interesting, as it could indicate that s5 is generated by a mechanism different than that responsible for s1.

The onset latency (Figure 6B) is a fundamental descriptor of neuronal responses and has been studied in several works (Phillips and Irvine, 1981; Phillips et al., 1985; Foeller et al., 2001; Hagemann et al., 2010; Hechavarria et al., 2013). For the primary sink s1 and the secondary sink s3, we found that significantly longer onset latencies could be elicited by low level stimulation (median = 21 ms, 64.6 ms) in comparison to CF80 (median = 14.6 ms, 50.8 ms). That latency decreases with stimulus amplitude is a common feature, which is already present at the level of the auditory nerve as described in several studies (Picton et al., 1974; Phillips and Irvine, 1981; Polich et al., 1988; Mendelson et al., 1997). The two stimuli spectrally distant from the CF (−1oct and +1oct) did not follow the same trend, although sharing the same octave distance. While the s1 and s3 obtained at −1oct stimulation were significantly different to s1 and s3 obtained during CF24+ stimulation, the median of s1 and s3 showed a non-significant latency shift of 3.6 and 8.4 ms during +1oct stimulation, which corresponds to previous studies (Kaur et al., 2005; Happel et al., 2010). The latencies of s5 and s8 during CF24+ stimulation had similar values in comparison to CF80, −1oct, and +1oct stimuli, although the variance was high. In all four groups, sink s1 [likely elicited by inputs from the ventral division of the geniculate body (MGv)] showed longer latencies [median s1–s2: 3.2 ms (CF80); 3.2 ms (−1oct); 6.5 ms (+1oct); 3.7 ms (CF24+)] in comparison to s2 (see Supplementary Figure 1B), which is thought to be created by the medial division of the geniculate body (MGm; Linden and Schreiner, 2003).

The duration of primary sink s1 (Figure 1C) showed no significant differences between the four stimulus groups, but was longer than the duration of the phasic-like activation of neuronal spikes which were about 19 ms for CF80, −1oct, and +1oct stimuli and about 14 ms for CF24+ stimuli (measured as the duration of suprathreshold spikes at 3.5 times the standard deviation beneath the baseline). Differences at the level of significance (*p* < 0.05) were observed for s3 and s5, both with opposite trends. While the duration of s3 was significantly shorter for CF24+ (median = 202 ms) than for +1oct (median = 258 ms), the duration of s5 was significantly longer for CF24+ (median = 253 ms) than for CF80 (median = 133 ms) and +1oct (median = 163 ms). The same trend could be observed for s8 but the differences were not significant.

The vertical extent (Figure 6D) revealed a homogeneous pattern for primary sink s1 and secondary sink s3 and s8, in which low level elicited sinks (median = 457 μm, 404 μm, 258 μm) had the significantly shortest vertical extents in comparison to CF80 (median = 504 μm, 504 μm, 357 μm). While s1, s3, and s8 of +1oct (median = 498 μm, 474 μm, 304 μm) followed the same trend, it was only at the level of significance for s8. Interestingly, the vertical extent of sink s5 appeared to be unaffected by different stimuli and showed no significant differences similar to the maximum strength or onset latency.

### Correlation of Laminar CSD Profiles

**Figure 7:**
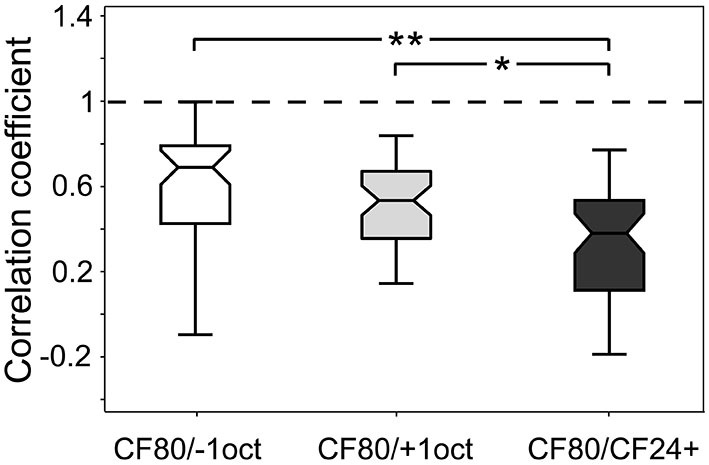
**Stimulus-specific correlation of laminar CSD profiles.** Boxplots show the correlation coefficient between the laminar profiles of CF80 compared to the remaining stimuli (−1oct, +1oct, and CF24+). Boxplot whiskers represent data range, outer edge of box represents second and fourth quartiles of data, and midline represents median of data. Significance was determined using Kruskal-Wallis one-way ANOVA in combination with a multiple comparison *post hoc* test: *p < 0.05, **p < 0.01.

Significant stimulus-specific differences in the CSD patterns could be found at the level of sink parameters (see Figure 6). To be able to account for the overall difference of laminar patterns, including sinks, sources and background noise, we also calculated correlation coefficients between CSD patterns obtained for all different stimuli compared to the control CF80. The latter was done for each recording tract separately. In Figure 7 the results are displayed as boxplots. The correlation between CF80 and −1oct or +1oct was significantly higher (*r* = 0.69 ± 0.3; *r* = 0.53 ± 0.3) than the correlation between CF80 and CF24+ (*r* = 0.38 ± 0.25). Most significant differences in the sinks (see Figure 6) could be observed for the low level CF24+ stimulus, which also yields the weakest correlation with the response obtained with CF80 stimuli.

## Discussion

### Methodological Considerations and Limitations

LFP are influenced by nonspecific factors such as ongoing cortical activity (spontaneous or evoked by a preceding stimulus) or the state of excitability (Mitzdorf, 1987). Most anesthetics appear to decrease neural conduction and synaptic transmission, thereby decreasing the amplitude and increasing the latency of evoked responses (Armstrong-James and George, 1988; Kuwada et al., 1989; Zurita et al., 1994; Sloan, 1998; Gaese and Ostwald, 2001). Considerable contribution to variances of CSD patterns could come from up and down states, which are associated with different states of cellular excitability that effectively influences evoked potentials by generating either increased or decreased amplitudes (Timofeev and Steriade, 1998; Petersen et al., 2003; Saleem et al., 2010). Such effects may be stronger for mid (Mitzdorf, 1987) and long latency sinks as many sources could contribute to their generation.

The CSD technique provides insights into the activation patterns of ensembles of cortical neuronal populations at sublaminar dimension, but this method employs a number of assumptions, such as isotropic current in the tangential direction and uniform conductivity, neither of which is strictly accurate for the cerebral cortex. Aside from this, Tenke et al. (1993) pointed out that the accuracy with which the CSD analysis can be related to the activation of underlying neural elements is influenced by noise and spatial resolution. The use of uniformly manufactured commercially available multicontact electrodes utilized in the present study allowed us to minimize interchannel distance inaccuracies. The temporal sampling resolution is crucial for adequately representing activity alterations having a high frequency, whereas the spatial sampling resolution is important for gathering detailed information on subpopulations of participating neurons. In the present study, the temporal resolution was much higher (20 kHz) than that required for LFP measurements whose frequencies are defined to be below 300 Hz. The spatial resolution (channel spacing of 100 μm), which proved useful in previous studies in rats (Kaur et al., 2005; Szymanski et al., 2009), was supposed to be adequate to identify functional interactions that proceed within individual cortical laminae or sublaminae, but could also magnify the contributions of computational artifacts (Tenke et al., 1993). However, CSD patterns are superpositions of multiple corresponding sinks and sources. At longer latencies, a larger number of sources and sinks can interfere, which makes it increasingly difficult to separate them. Therefore late sinks do not necessarily reflect a “true sink component” in the sense of a mass trans-membrane current at a certain location and latency and should be considered critically.

### The General Structure of CSD Flow-Patterns in the Mammalian Neocortex

The more or less uniform neuronal architecture of the neocortex and its six-layered structure corroborate the hypothesis that afferent activity is relayed very similarly in all sensory areas and reflects the same types of excitatory synaptic ensemble activities along the same intracortical pathways thus leading to similar CSD patterns (Atencio and Schreiner, 2010). Several studies in the visual cortex of monkeys (Mitzdorf and Singer, 1979; Kraut et al., 1985), cats (Mitzdorf, 1985, 1987), and rabbits (Rappelsberger et al., 1981; Pockberger and Rappelsberger, 1983), in the somatosensory cortex of rats (Di et al., 1990) or in the auditory cortex of monkeys (Müller-Preuss and Mitzdorf, 1984; Steinschneider et al., 1992), rats (Kaur et al., 2005; Szymanski et al., 2009) or gerbils (Happel et al., 2010) led to similar patterns where early sinks (s1 and s2) were present in the input layer IV [and deep layer III in the somatosensory or auditory cortex (Huang and Winer, 2000; Lee and Winer, 2008)] and in many studies [mostly in the auditory cortex (Mitzdorf, 1985)] at the layer V/VI border. Later mid latency sinks in layer II, V, and VI were apparent in most of these profiles with a considerable delay and strongly resemble s4, s5, and s3 of the present study (Pockberger and Rappelsberger, 1983; Mitzdorf, 1985, 1987; Di et al., 1990; Lakatos et al., 2007; Happel et al., 2010; Kajikawa and Schroeder, 2011). However, qualitatively different CSD patterns were found in the visual (Lakatos et al., 2009) and auditory cortex (Lakatos et al., 2007, 2009; Kajikawa and Schroeder, 2011; O’Connell et al., 2011; Tenke and Kayser, 2012) of monkeys, where the activation sequence in layers II–IV was intermediated by a source in layer III. Architectonical differences in the laminar structure of the monkey cortex, indicated by differently proportionate layers and concurrent broadening of supragranular and compression of infragranular layers (Lakatos et al., 2005; Maier et al., 2011; O’Connell et al., 2011) could serve as an explanation. There are no other studies on late sinks s6–s9 available for the auditory cortex. In the visual cortex of cats late sinks were present in layers III, V, and VI, which could resemble s8, s6, and s7 of the present study, but showed far longer onset latencies (Mitzdorf, 1985, 1987).

### Neuronal Origins of Early Evoked Sinks

In the present study, s1, located in the input layers III and VI, likely reflects lemniscal thalamocortical input from tonotopically organized afferent inputs from the ventral part of the medial geniculate body (MGv; Budinger et al., 2000; Huang and Winer, 2000). Small pyramidal cells in layers IIIb and IV seem to be the main thalamorecipient neurons in auditory cortex (Linden and Schreiner, 2003; Wallace and Palmer, 2008). Experiments on silencing cortex by the GABA_A_ agonist muscimol further confirmed the contribution of cortical neurons on the generation of s1 (Kaur et al., 2004; Happel et al., 2010). Sink s2, located at the layer V/VI border, was present in almost every CSD pattern (see Figure 5) and likely reflects non-lemniscal thalamocortical input from the medial part of the medial geniculate body (MGm; Steinschneider et al., 1998; Kimura et al., 2003; Linden and Schreiner, 2003; Winer and Lee, 2007). These deep initial sinks had shorter onset latencies [11.4 ms (see Supplementary Figure 1)] than the sinks in infragranular layers III–IV (14.6 ms), which was in accordance with previous studies in the primary auditory cortex of rodents [rat (Kaur et al., 2005; Szymanski et al., 2009), gerbil (Sugimoto et al., 1997), mouse (Shen et al., 1999), and guinea pig (Wallace and Palmer, 2008)].

### Neuronal Origins of Mid and Late Evoked Sinks

To our knowledge, no quantitative study thus far has characterized the late sinks (s6–s9). While the origins of the early initial sinks are known to a large degree, those of the successive mid and late cortical activations are rather obscure and proposed to be composed of an ensemble activity generated by different areas from the primary and secondary cortical fields, the FC, the centro-parietal cortex, association cortex, and/or the hippocampus (Picton et al., 1974; Skrebitsky and Sharonova, 1976; Kraus et al., 1985; Mitzdorf, 1985; Näätänen and Picton, 1987; Barth and Di, 1990; Santos Filha and Matas, 2010). With our current data we cannot resolve the neuronal origins of mid and late evoked sinks. Although mid and late components might have an important role in the cortical processing, as they are more or less reliably evoked. It is difficult to speculate about the sources of these sinks, as the temporal gap between initial excitatory and delayed activity is approximately 50 ms (comparing the onset latencies). This represents a relatively long cortical processing time during which many possible mechanisms could operate. Di et al. (1990) proposed that the later components in the CSD patterns of the rat barrel cortex represent hyperpolarizations or repolarizations. However, several other studies assumed different mechanisms for mid and late sinks assignable to at least three categories of synaptic input: (1) repetitive after-discharges from thalamus relay neurons; (2) intrinsic processing within the AC microcircuits; and (3) inputs from outside of the AC such as the FC, the contralateral hemisphere or the hippocampus. We will discuss these possibilities in the same order as depicted above.

**Figure 8:**
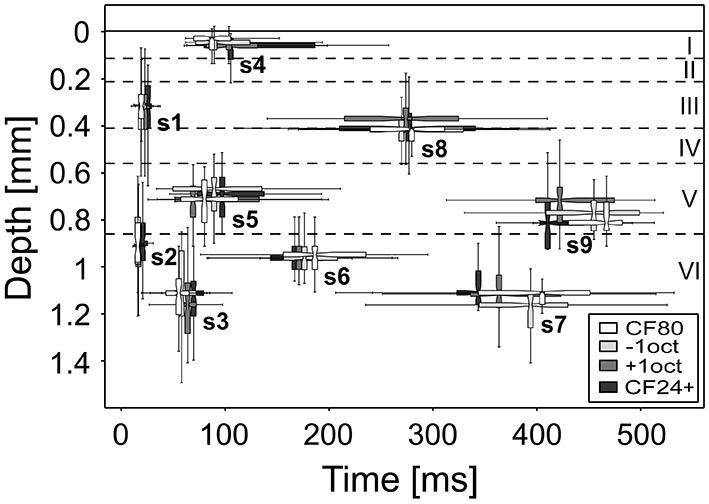
**Onset latency and depth of stimulus-specific sinks.** The range of the onset depth is displayed at the median of the respective onset latency. The sinks remain relatively constant regarding the layer and time at which the neuronal activity is evoked. Boxplot whiskers represent data range, outer edge of box represents second and fourth quartiles of data, and midline represents median of data.

**Figure 9:**
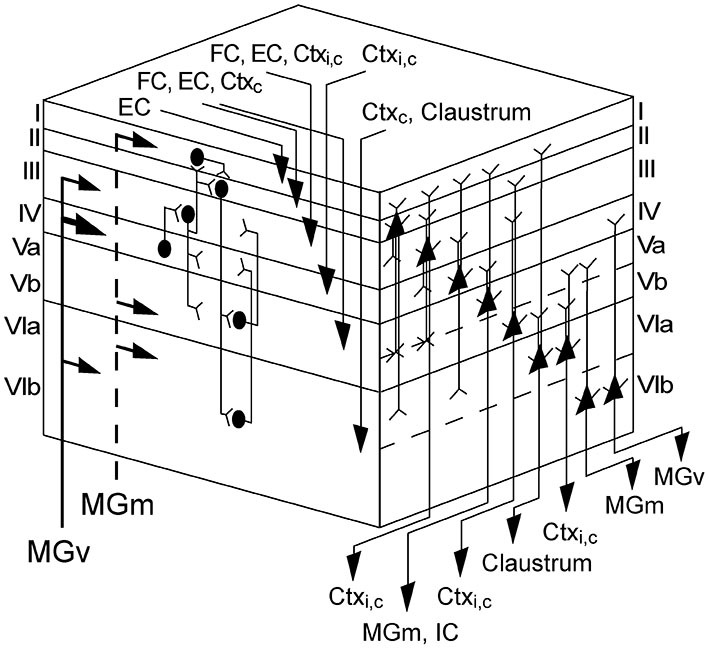
**Cortical and laminar network organization of the AC.** Left face of cube shows inputs from the ventral (MGv) and medial divisions (MGm) of the geniculate body and cortical areas and a schematic of the interlaminar connections; right face displays the pyramidal neurons of each layer and gives their axonal targets. Note that this schematic drawing illustrates only the major pyramidal neurons. Lemniscal thalamic inputs from MGv mainly arrive at layers III and IV, but also at layer VI. Nonlemniscal inputs from MGm activate layers I, Vb, and VIa. Thalamic activation of layer IV initiates a flow of information into the supragranular layers I–III and then down to the infragranular layers V and VI. Corticocortical inputs from the ipsilateral hemisphere (Ctx_i_) terminate in the middle layers III and IV. Commissural inputs (Ctx_c_) are widely distributed and arrive in almost all layers (II–VI). Inputs from the claustrum terminate in layer VI, whereas the entorhinal cortex (EC) and frontal cortex (FC) projects to neurons in layers (I) II, III, and V. Pyramidal cells are present in all layers, but layer I. The pyramidal axons of layers II–VI extend into layer I, with the exception of different pyramidal cell types in layers VIa and VIb. Corticocortical projections emerge from layers III, V, and VI. Feed-back to the auditory thalamus originates primarily in layer VI, but also in layer V, which also projects to the inferior colliculus (IC). Adapted from Mitani et al. (1985), Insausti et al. (1997), Linden and Schreiner (2003), Mitchell and Macklis (2005), Thomson and Lamy (2007) and Izhikevich and Edelman (2008).

(1) Repetitive thalamocortical after-discharges as a generator of mid and late sinks has been described in the visually cortex of rabbits (Rappelsberger et al., 1981) and cats (Mitzdorf, 1985). They occurred rather consistently when the animal was deeply anesthetized and/or its neurons exhibited low excitability. Mitzdorf (1985) observed that these after-discharges usually induce only small sinks in the input layers and usually recurred a few times with successively lower amplitudes and longer intervals. It has been suggested that these after-discharges are generated in the thalamus (Buser and Horvath, 1972; Horvath and Buser, 1972) or in the retina in the case of the visual cortex (Wachtmeister and Dowling, 1978) and are conducted to the cortex via specific afferents. Taken the thalamocortical wiring into account, in which MGv projects to layers III, IV, and VIb and MGm to layers I, Vb, and VIa, most of the sinks (s3, s4, s6, s7, and s8) could be explained by comparing the thalamocortical target layers with the cortical depth of the earliest activity within a sinks, in short onset latency depth (Figure 8). As pyramidal neurons in layers Va and VIb are the main sources of thalamic feed-back projections (Linden and Schreiner, 2003), s5 and s7 both having similar maximum strength could originate from reciprocal inputs (Figure 9). However, pyramidal neurons are not exclusively activated by thalamocortical inputs as they form connections to many different ipsi- and contralaterally located non-thalamic neurons (Mitani et al., 1985; Linden and Schreiner, 2003; Thomson and Lamy, 2007; Izhikevich and Edelman, 2008).

(2) The contribution of intrinsic cortical connections could also serve as an explanation for mid and late evoked sinks. Kaur et al. (2004) could show a partial suppression of initial and a full suppression of longer-latency response components with muscimol, suggesting a major involvement of intracortical pathways. However, it is not clear to what extent activity from the primary auditory cortex contributes, directly or indirectly to mid or late evoked sinks. It is known that the majority of synapses in a cortical column are intrinsically connected and less innervated by thalamic or distant cortical areas (Douglas and Martin, 2007). Five to twenty percent of the inputs to the granular layer IV originate from convergent thalamocortical and 80–95% from intracortical projections (Peters et al., 1994; Ahmed et al., 1997; Logothetis, 2008). It is known that intrinsic vertical and horizontal connections (Figure 9) influence the cortical network (Mitani et al., 1985; Matsubara and Phillips, 1988; Read et al., 2001; Atencio and Schreiner, 2010) and could thus dominate the generation of later sinks. But it is still unclear if the intracortical synapses are related to local intracolumnar or intercolumnar neurons, as it has been proposed based on anatomical data (Ojima et al., 1991, 1992; Budinger et al., 2000). In addition, there are parallel excitatory and inhibitory circuits (feed-forward and feed-back) in which different inhibitory interneurons play an important role in the regulation of cortical operations (Shepherd and Koch, 1998; Kanwal et al., 1999) that could further contribute to the shaping of late sinks. However, the contribution of inhibitory postsynaptic potentials to the sinks is at least one order of magnitude smaller in amplitude than the contribution of excitatory postsynaptic potentials (Mitzdorf, 1987; Schroeder et al., 1990; Tenke et al., 1993).

(3) Ferreyra-Moyano et al. (1988) demonstrated for the rat olfactory cortex that activation of pyramidal neurons from outside of the AC may be the main generators of late evoked components, rather than re-excitation of mitral cell axons. Certain types of secondary responses associated with complex brain functions are influenced by barbiturate resulting in either reduced or abolished activity (Fuster and Docter, 1962; Torres and Warner, 1962). These responses are correlated with activation of the mesencephalic reticular formation (Fuster and Docter, 1962; Torres and Warner, 1962) and are related to memory (John, 1967) and passive or active conditioning (Sakhiulina and Merzhanova, 1966; Fox, 1970). It is also believed that the FC, the centro-parietal cortex, and the hippocampus are possible generators for late components (Santos Filha and Matas, 2010). Evidence for an important role of mid latency sinks in somatic sensory perception has been suggested by a previous work which showed that depolarizations predicted the sensory discrimination performance of monkeys (Kulics and Cauller, 1986). In addition, using auditory and visual oddball tasks, Halgren et al. (1998) reported that sensory-specific areas have a long period of activity to which widespread brain circuits contribute at approximately the time of late sinks onset (s6–s9). The above mentioned circuits are related to the paralimbic and attentional frontoparietocingular cortex and to the event-encoding cortices, the association cortex, and the hippocampus. Neurons from the hippocampus project via the entorhinal cortex (EC) mainly to neurons in superficial layers I, II, and III and infragranular layer V of sensory cortices (Kosel et al., 1982; Insausti et al., 1997). As hippocampus and EC are related to spatial memory, navigation, and episodic memory (Jacobs et al., 2010), it is likely to assume, that repetitive stimulation can trigger neuronal activity in both areas contributing to the generation of late evoked sinks s8 and s9. Similar to the EC, the FC also targets layers II, III, and V (Mitchell and Macklis, 2005). It has long been suspected to play an important role in cognitive control, in the ability to determine actions in accordance with internal goals (Fuster, 2000; Kanwal et al., 2000; Sakamoto et al., 2015). Research based on surface recordings indicated that long-latency auditory evoked potentials probably arise from frontal associative areas (Picton et al., 1974; Iwasa and Potsic, 1982). As sink s8 showed a rather strong activation compared to most of the later sinks (s5, s6, and s7), it could be evoked by a convergent interaction of several different brain areas such as the hippocampus or the FC. However, its reliability could indicate a stable source of generation e.g., thalamic feed-back projections to layer IV which would imply a more or less constant time delay between both sinks s1 and s8 (s5). But this is not the case as the time delay shortens in dependence of applied frequency or level (Figure 8) due to a latency shift in s1. Late sink s7 located in layer VIb could be evoked by inputs from contralateral cortices and/or the claustrum as suggested by cortical wiring (Linden and Schreiner, 2003; Thomson and Lamy, 2007). The fact, that s3, s4, s6, and s8 have a considerably longer duration than s5, s7 or s9 (see Supplementary Figure 1) could mean, that they reflect modulatory output.

In summary, we can propose a hypothetical scheme of the specific sink generators based on results of previous studies and on the cortical architecture. The early part of s1 and s2 is clearly of thalamic origin and the later part with a high possibility of intrinsic cortical origin. The mid latency sinks s3–s5 are most likely evoked by inputs from cortical areas due to the long latency. The late sinks s6–s9 are supposedly evoked by specific brain areas such as the frontal or EC, which are temporally correlated with these sinks (see Figure 8) and are associated with memory, learning or cognitive control.

### Change of Current-Flow-Patterns Due to Level and Spectral Integration

We investigated differences in the CSD patterns, especially for the mid and late sinks, in relation to three different stimuli residing at the frequency and sensitivity corners of respective neuronal receptive fields. Our results show that the intracortical information-flow-patterns do not exhibit qualitative changes in response to the stimuli tested. Several studies proposed that short-range or long-range intracortical (“horizontal”) connections could provide spectral input (excitatory and inhibitory) to differently tuned columnar neurons receiving spectrally different direct thalamic input (Kaur et al., 2004, 2005; Tomioka et al., 2005; Kurt et al., 2008; Happel et al., 2010; Moeller et al., 2010). Other studies demonstrate large scale spectral integration based on the receptive fields of single neurons (Kanwal et al., 1999) and assume that spectral information within the range of CF ± 1 octave is fed through direct thalamocortical projections to layer III/IV (Kaur et al., 2005; Happel et al., 2010; Guo et al., 2013). It has been shown that the laminar organization of horizontal and thalamocortical inputs are different in a way that stimulation near the frequency eliciting the highest firing rate led to activations of layer IIIa (Happel et al., 2010). This is in accordance with the excitatory horizontal inputs mainly terminating in layer II/III (Dantzker and Callaway, 2000; Thomson and Bannister, 2003). We could not observe this shift of activity in our dataset, as the cortical depth of the maximum strength within s1 was constantly located in layer III and the vertical extent remained similar for CF80 and near CF stimuli (see Supplementary Figures 1D,F). In regard to s8 located in layer III/IV we could observe a quite reliable shift in the depth of the maximum strength towards layer III for +1oct and CF24+ stimuli (Figure 5) indicating a contribution of horizontal inputs. Most of the initial, mid, and late sinks showed partly significant differences such as maximum strength, onset latency, duration, and vertical extent already at a spectral distance of CF ± 1 octave (Figures 6A–D). Stimulation with +1oct produced a characteristic laminar activation profile in the initial granular sink s1 with (non-significantly) decreased maximum strength and an increased onset latency (and duration; Figures 6A–C), which was in accordance with previous works (Kaur et al., 2005; Happel et al., 2010; O’Connell et al., 2011). In comparison to CF80 stimulation the onset latency of s1 and s3 during +1oct stimulation increases in contrast to s5 or s8 (Figure 6B), supposing s3 as a direct or indirect (via s1 and s2) target for spectral integration fibers initializing a feed-forward activation cascade (Happel et al., 2010; Guo et al., 2013). In contrast to this, the same spectral distance from the respective CF in the opposing direction (−1oct) produced initial sinks (and to a certain degree also mid and late sinks) which resemble the ones of CF80 stimulation in terms of maximum strength, onset latency and vertical extent (Figures 6A,B,D, 8). This could indicate that both +1oct and CF24+ follow comparable processing mechanisms or at least activate similar neuronal clusters. Discrepancies in the onset latencies evoked by spectrally different stimuli could also be observed by Guo et al. (2013). In contrast to our data, in the Guo study both spectrally distant CF ± 1 octave stimuli elicited longer s1 onset latencies of which +1oct (~+5 ms) is similar to our data (+3.6 ms) but not the −1oct (~+6.5 ms vs. –0.2 ms). However, both results are hard to compare as different sound levels (80 dB SPL vs. ~30–35 dB SPL) were used, but this could indicate a level dependent mechanism of spectral integration.

When comparing the whole CSD pattern, the tendency of −1oct eliciting similar sink parameter as CF80 is reflected by a higher correlation coefficient (*r* = 0.69 ± 0.3) than for CF80 and +1oct (*r* = 0.53 ± 0.3, Figure 7). One reason for a discrepancy between two equally spectrally distant stimuli could originate from asymmetrically shaped tuning curves. This shifts +1oct stimuli to a higher percentage (66%) outside of the receptive field in contrast to −1oct (96%, Figure 3D). Asymmetrically V-shaped tuning curves are a consequence of cochlear mechanics (Zwislocki, 1983; Kössl et al., 2003; Vater and Kössl, 2011) and found in many species at the cortical level (Kanwal et al., 1999; Foeller et al., 2001; Polley et al., 2007; Hoffmann et al., 2008). In addition, cortical tuning curves are modified by interaction and convergence of afferent, local, and long-range intracortical inputs (Happel et al., 2010) and different synaptic populations could be active during shaping of the respective flanks of receptive fields. As intracortical feed-forward inhibitory circuits were recently suggested to be responsible for lateral sharpening of spectral tuning (Kanwal et al., 1999; Wu et al., 2008), the high frequency flank could be more affected by this circuit activity leading to a sharper flank and thus to different CSD patterns. A relatively clear evidence for asymmetrical processing of spectrally distant stimuli of the same distance to the CF (± 1 octave) can be provided by the relatively reliable appearance (68%) of an additional late sink s9 in +1oct patterns (Figure 5). The fact that s9 can also be elicited with a low probability of ~20% with the remaining stimuli suggests that a basic cortical pathway is involved which enhances the appearance of s9 in response to certain stimuli.

Taking the cortical wiring (Figure 9) and the large onset latency distance to initial sinks (~400 ms) into account (Figure 8), s9 is probably generated by different areas such as the FC, EC or the contralateral hemisphere. Reasons why frequencies one octave above the CF would evoke s9 remain speculative. But for the perception of communication calls which commonly consist of several harmonics (Medvedev and Kanwal, 2008; Kobayasi et al., 2012) a sink coding for higher frequencies would be of behavioral advantage as it could provide additional activity alongside s5 in layer V leading to subsequent behavior related areas being activated over a longer time period. The extent of synaptic activity was significantly reduced for CF24+ stimuli compared to CF80 while onset latency depth remained unchanged (Figure 8). The observed delayed onset latency for CF24+ could have another origin which is probably related to the temporal integration of the pressure envelope of the sound in the auditory periphery (Heil and Neubauer, 2001, 2003; Heil et al., 2008). These auditory nerve results were all based on neuronal spike data. Most of the sinks (Figure 6) elicited by CF24+ stimulation showed significantly reduced neuronal activity and longer onset latencies in comparison to higher level stimulation which is in accordance with a previous study concerning initial sinks (Lakatos et al., 2007). The maximum strength, onset latency, and vertical extent of s5 in layer V were unaffected by both spectral distance and level of the stimuli. Layer V integrates inputs from different cortical areas and projects back to the thalamus (Figure 9) and contains a high concentration of D1 receptors, which were suggested to modulate memory formation (Schicknick et al., 2008; Scheich et al., 2011). This could imply that s5 is a result of general modulatory actions. Interestingly, each of the mid and late sinks evoked by CF24+ was less reliably evoked than during high level stimulation. And sink s7 which during high level stimulation showed one of the largest deviations in terms of onset latency and depth (Figure 8) is quite reliably missing for CF24+ stimuli (61%). This emphasizes that the appearance of s7 as well as the remaining mid and late sinks are dependent on the level of stimulus.

## Concluding Remarks

Our results provide a baseline for studies where precise quantitative physiological data are needed for modeling neuronal circuits involving different subcortical, intracortical, and corticocortical areas. The stimulus-specific differences in the sink metrics as well as the missing sink s7 and s9 support the hypothesis that the auditory cortex processes stimuli differently according to their frequency and level content. This could originate from stimulus-specific proportioned spatial and temporal interactions and convergences potentially leading to different processing patterns. Comparing the depth aligned onset latencies, which provide information about the sink specific laminar origins of the initial relay clusters (Figure 8), sinks s2 and s6 as well as s1 and s8, s3 and s7 or s5 and s9 (referring to s9 elicited by +1oct as the others stimuli provide an insufficient data situation) could originate from common neuronal clusters. This would mean that stimulus processing is maintained by an activity of five main relay stations within layers I, III, Va, VIa, and VIb. Future studies on mid and late evoked sinks should focus on these layers for cortical silencing or electrical stimulation to learn more about their origins and processing mechanisms.

## Conflict of Interest Statement

The authors declare that the research was conducted in the absence of any commercial or financial relationships that could be construed as a potential conflict of interest.
