# Supplementary material for: Quantification of mid and late evoked sinks in laminar current source density profiles of columns in the primary auditory cortex
Source: Front Neural Circuits. 2015 Oct 2;9:52. doi: 10.3389/fncir.2015.00052 (PMC4617414; doi:10.3389/fncir.2015.00052)
Supplement: Supplementary file 1 [file DataSheet_1.doc]

***Supplementary Material***

**Quantification of mid and late evoked sinks in laminar current source density profiles of columns in the primary auditory cortex**

***Markus Schaefer*, Julio C. Hechavarría and Manfred Kössl***

Institute for Cell Biology and Neuroscience, AK Neurobiologie und Biosensorik, Goethe University, 60438 Frankfurt/Main, Germany

***Correspondence:**

Markus Schaefer

Institute for Cell Biology and Neuroscience

AK Neurobiologie und Biosensorik

Goethe University

Max-von-Laue-Str. 13

60438 Frankfurt/Main, Germany

markus.sbio@web.de


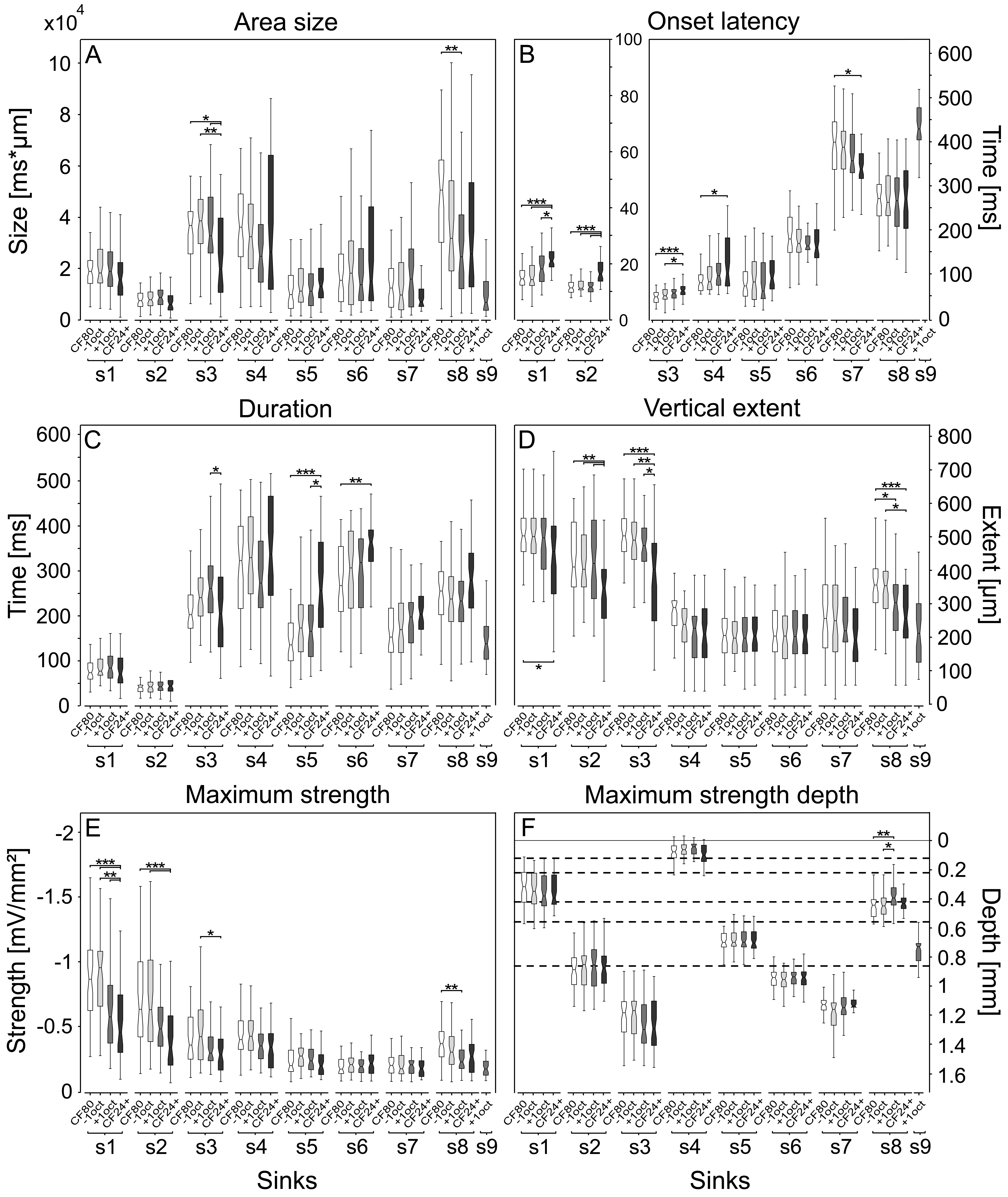


**SUPPLEMENTARY FIGURE 1 | Quantitative comparison of sink parameters.** Parameters were calculated at 8% of the maximum sink strength of the respective CSD pattern elicited by CF80 stimulation. Low level pure tones (CF24+) and pure tone frequencies of one octave distant from the CF (‑1oct and +1oct) evoke significantly different sinks. **(A)** The sink area size highly varies across sinks. **(B)** The onset latency for s2 is significantly shorter than for s1. Onset latency of s1‑s4 is significantly longer for low level stimulation, but significantly shorter for s7. **(C)** Compared to CF80 stimulation the sink duration of s4‑s8 is increased for CF24+ stimulation. Sink s3 shows the significantly shortest sink duration compared to +1oct. **(D)** The vertical extent shows a relatively comparable pattern for s1‑s3, s7, and s8 in which CF24+ mostly elicits significantly lower vertical extents in comparison to CF80 and ‑1oct. **(E)** The maximum strength of sinks s1‑s4 and s8 elicited by ‑1oct and CF80 is mostly, significantly higher than for +1oct and CF24+. Highest values are found in the thalamic input layers III/IV (s1) and V/VI (s2). **(F)** The vertical position of the maximum strength which could indicate projections to different population of neurons is only significantly different for s8. Sink s8 elicited by +1oct (and CF24+) has its highest strength significantly shifted to lower layer III in comparison to CF80 and ‑1oct. Boxplot whiskers represent data range, outer edge of box represents second and fourth quartiles of data, and midline represents median of data. Significance was determined using Kruskal-Wallis one-way ANOVA in combination with a multiple comparison post-hoc test: *p<0.05, **p<0.01, ***p<0.001.
